# Father and Son

**Published:** 1914-07

**Authors:** Ella Wheeler Wilcox


### Father and Son.

My grand-dame, vigorous at eighty-one,
Delights in talking of her only son,
My gallant father, long since dead and gone.
"Ah, but he was the lad!"
She says, and sighs, and looks at me askance.
How well I read the meaning of that glance—
"Poor son of such a dad."
Poor weakling, dull and sad."
I could, but would not, tell her bitter truth
About my father's youth.

She says: "Your father laughed his way through earth;
He laughed right in the doctor's face at birth—
Such joy of life he had, such founts of mirth.
Ah, what a lad was he!"
And then she sighs. I feel her silent blame,
Because I brought her nothing but his name,
Because she does not see
Her worshiped son in me.
I could, but would not, speak in my defense
Anent the difference.

She says: "He won all prizes in his time.
He overworked, and died before his prime.
At high ambition's door I lay the crime.
Ah, what a lad he was!"
Well, let her rest in that deceiving thought.
Of what avail to say, "His death was brought
By broken sexual laws,
The ancient sinful cause."
I could, but would not, tell the good old dame
The story of his shame.

I could say: "I am crippled, weak and pale,
Because my father was an unleashed male.
Because he ran so fast, I halt and fail.
(Ah, yes, he was the lad!)

Because he drained each cup of sense-delight
I must go thirsting, thirsting, day and night.
Because he was joy-mad,
I must be always sad.
Because he learned no law of self-control,
I am a blighted soul."
Of what avail to speak and spoil her joy.
Better to see her disapproving eyes,
And, silent, hear her say, between her sighs,
"Ah, but he was the boy!"

—*Ella Wheeler Wilcox.*

